# Prevalence and associated factors of early initiation of sexual intercourse among youth in Ethiopia: systematic review and meta-analysis

**DOI:** 10.1186/s12889-023-16968-y

**Published:** 2023-10-23

**Authors:** Natnael Kebede, Fekade Demeke Bayou, Fanos Yeshanew Ayele, Bereket Kefale, Asnakew Molla Mekonen, Anteneh Mengist Dessie, Yawkal Tsega

**Affiliations:** 1 Department of Health Promotion, School of Public Health, College of Medicine and Health Sciences, Wollo University, Dessie City, Ethiopia; 2 Department of Public Health, College of Medicine and Health Sciences, Wollo University, Dessie City, Ethiopia; 3 Department of Public Health Nutrition, College of Medicine and Health Sciences, Wollo University, Dessie City, Ethiopia; 4 Department of Reproductive and Family Health, School of Public Health, College of Medicine and Health Sciences, Wollo University, Dessie City, Ethiopia; 5 Department of Public Health, College of Health Science, Debre Tabor University, Debre Tabor, Ethiopia; 6 School of Public Health, College of Medicine and Health Sciences, Wollo University, Dessie City, Ethiopia

**Keywords:** Early initiation of sexual intercourse, Youth, Systematic review, Meta-analysis, Ethiopia

## Abstract

**Background:**

Early sexual initiation refers to engaging in sexual activity at a young age, typically before the age of 18. Even though many studies have been conducted in Ethiopia, the result is inconsistent between studies. In the study area, the pooled prevalence and associated factors of early initiation of sexual intercourse among youth were not done before. Therefore, this study aimed to determine the pooled prevalence and associated factors of early initiation of sexual intercourse among Youth in Ethiopia.

**Methods:**

This study used a systematic review and meta-analysis of studies conducted from 2008 to 2022, in Ethiopia. The Preferred Reporting Items for Systematic Reviews and Meta-Analyses (PRISMA) guidelines were followed. PubMed, Cochrane Library, Hinari, and Google Scholar electronic databases were searched. The analysis was performed using STATA 17 software. Heterogeneity and publication bias were assessed using forest plots, I^2^_,_ Cochran’s Q statistics and Funnel plots, Egger test, and Begg rank tests respectively. Duval and Tweedie’s ‘trim and fill’ method was also performed to adjust the pooled estimate. Pooled analysis was conducted using the inverse-variance fixed-effects model.

**Results:**

A total of 10 articles were included in this systematic review and meta-analysis. The pooled prevalence of early initiation of sexual intercourse among youth in Ethiopia was 24.7% (95%CI: 10.4, 38.9). Being female (AOR = 3.57; 95% CI: 1.387, 5.743), having poor knowledge of HIV/AIDS prevention (AOR = 3.65; 95% CI: 1.981,5.309), alcohol use (AOR = 2.05; 95% CI: 1.415, 2.679), khat chewing (AOR = 3.03; 95% CI: 1.800, 4.254), Viewed pornographic film(AOR = 4.21, 95% CI: 2.135, 6.283), Cigarette smoking (AOR = 2.74; 95% CI: 2.102, 3.370) and Poor family controls (AOR = 4.39; 95% CI: 2.572, 6.199)were associated factors of early initiation of sexual intercourse.

**Conclusions:**

The pooled prevalence of early initiation of sexual intercourse among Youth in Ethiopia was high. Being female, poor knowledge of HIV/AIDS prevention, alcohol use, khat chewing, Viewing pornographic films, Cigarette smoking, and poor family controls were associated factors of early initiation of sexual intercourse. It is recommended that targeted interventions be put in place to address the high prevalence of early initiation of sexual intercourse among youth in Ethiopia. These interventions should focus on addressing the associated factors such as poor knowledge of HIV/AIDS prevention, alcohol use, khat chewing, viewing pornographic films, cigarette smoking, and poor family controls. It is important that these interventions are gender-sensitive and take into consideration the unique challenges faced by females in accessing sexual and reproductive health services.

## Background

Early sexual initiation refers to engaging in sexual activity at a young age, typically before the age of 18 [1]. Early initiation of sexual intercourse is a risk factor for teenage pregnancy and contracting HIV and other sexually transmitted infections (STIs) [2–4]. Individuals who engage in sexual activity at a young age tend to have an increased likelihood of engaging in high-risk sexual behaviors such as having multiple partners and a reduced likelihood of using condoms. Delaying sexual debut is the corner of HIV/STI prevention among young people [5]. Globally, the high prevalence of teenage pregnancies among young age girls in particular is a major public health concern for the majority of parents, guardians, and duty-bearers [6, 7]. Youth in Sub-Saharan Africa have been reported to engage in high rates of premarital sexual activity, as several studies have found [8]. In Ethiopia, 29% of youth had their first sexual intercourse before the age of 15 years old and 62% of them had their first sexual intercourse before the age of 18 years old. The median age at first sexual intercourse for women is 16.6 [9]. According to the 2016 EDHS, The median age at first sexual intercourse is 0.5 years earlier than the median age at first marriage for women and 2.5 years earlier for men; this indicates that both women and men engage in sex before marriage [10].

The timing of initial sexual activity is a matter of public health concern [11]. The occurrence of giving birth at a young age has been associated with increased rates of maternal and child illness and death, limited educational options, reduced family income in the future, and larger family sizes, which may ultimately result in higher population growth [12, 13] .in addition it consequences higher rates of unintended pregnancy, abortion, HIV/AIDS and other sexually transmitted infections among youths [14].

As indicated by various studies, Being female [15–20], residence [21–23], religion [6, 21–25], self and parental education [19, 22], socioeconomic status [6, 19, 22], alcohol use [16–19, 21, 22], khat chewing [16, 22, 24], Cigarette smoking [20], Viewing pornographic films at age < 18 years [16, 17, 24], visiting night or day party [17], classmate friend/peer pressure [18, 19, 23, 24], poor family controls [6, 16, 20, 25], positive attitudes regarding condom efficacy [21] poor knowledge of HIV/AIDS prevention [22] and more positive attitudes to family planning use [21] were the factors associated with early sexual initiation. So, it is crucial for a need to understand and assess the factors that are associated with early sexual initiation.

Even though many studies have been conducted in Ethiopia, the result is inconsistent between studies. In the study area, the pooled prevalence and associated factors of early initiation of sexual intercourse among youth were not done before. Assessing the pooled result will help to inspire governmental commitments and increase the mobilization of financial resources to enhance the implementation of evidence-based interventions to culminate the effect of early initiation of sexual intercourse among youth in particular and the nation in general. Therefore, this study aimed to determine the pooled prevalence and associated factors of early initiation of sexual intercourse among Youth in Ethiopia.

## Methods

### Study design and search strategy

The protocol of this systematic review and meta-analysis had been developed based on the Preferred Reporting Items for Systematic Reviews and Meta-Analyses Protocol (PRISMA) [26]. A systematic review and meta-analysis of published and unpublished studies were performed to assess the pooled prevalence and associated factors of Early Initiation of Sexual Intercourse among Youth in Ethiopia. Electronic databases such as PubMed, Cochrane Library, Hinari, and Google Scholar, were used. The following key terms were used to search studies: “prevalence”, “magnitude”, “proportion”, " Early Initiation of Sexual Intercourse “, " Sexual Intercourse “, “risky sexual behavior”, " sexual behavior “, “sexual desired”, “condom utilization”, and “HIV test”, “factors”, “determinants”, “predictors”, “factors associated”, “associated factors”, “risk factors”, “youth”, “College”, “students”, “high school students”, “undergraduate students”, “Ethiopia” by a combination of Boolean operators “AND” or “OR” as applicable and the search was made by three authors independently (NK, FDB, and FY).

### Inclusion and exclusion criteria

This review includes all accessible studies done from 2008 to 2022. All published and unpublished studies conducted on the prevalence and associated factors of early initiation of sexual intercourse among youth in Ethiopia were incorporated in the review. All observational studies with English language publications that measured the prevalence of Early Initiation of Sexual Intercourse among Youth in Ethiopia were considered in this review. However, irretrievable from the internet or those who have not received an email reply from the corresponding authors and studies with poor methodological quality were omitted from the review.

### Study selection, quality appraisal, and data extraction

All articles explored from selected databases were exported to Endnote X20 and duplicate files were dropped. Three investigators (NK, AMD, and YT) screened the leftover articles and abstracts for inclusion in the full-text appraisal. The difference between reviewers was managed through discussion, and disagreement was handled by the third party (AMM). The Joanna Briggs Institute (JBI) critical appraisal checklist for the prevalence study was used to evaluate the quality of articles that fulfilled the inclusion criteria [27]. Two reviewers independently assessed articles before inclusion in the review. Articles with quality scores of fifty and above were considered in the final review.

Microsoft Excel 2013 sheet was used for data extraction. The information on the author’s name, year of study, study design, response rate, sample size, study quality score, and prevalence were contained in the data extraction tool.

### Statistical methods and analysis

Data were analyzed using STATA 17 software. The pooled odds ratio (OR) with 95% CI was determined to estimate the determinants of Early Initiation of Sexual Intercourse among Youth. Pooled analysis was conducted using the inverse-variance fixed-effects model since there was no evidence of detected heterogeneity. The existence of heterogeneity among studies was examined by the forest plot, Cochrane’s Q test, as well as the I² heterogeneity test. The Cochrane Q statistic was considered significant if the p-value is < 0.10. While the ***I***^***2***^ statistics of at least 50% were considered to be significant [28]. Funnel plots analysis, Egger weighted regression, and Begg rank correlation tests were done to detect publication bias (P < 0.05 was considered suggestive of statistically significant publication bias) [27, 29]. Moreover, Duval and Tweedie’s ‘trim and fill’ method was used to estimate the number of missing studies from the meta-analysis [30].

### Registration and reporting

This systematic review and meta-analysis were registered in the PROSPERO with a CRD number of 42,023,413,767.

## Results

### Study selection

**Fig. 1:**
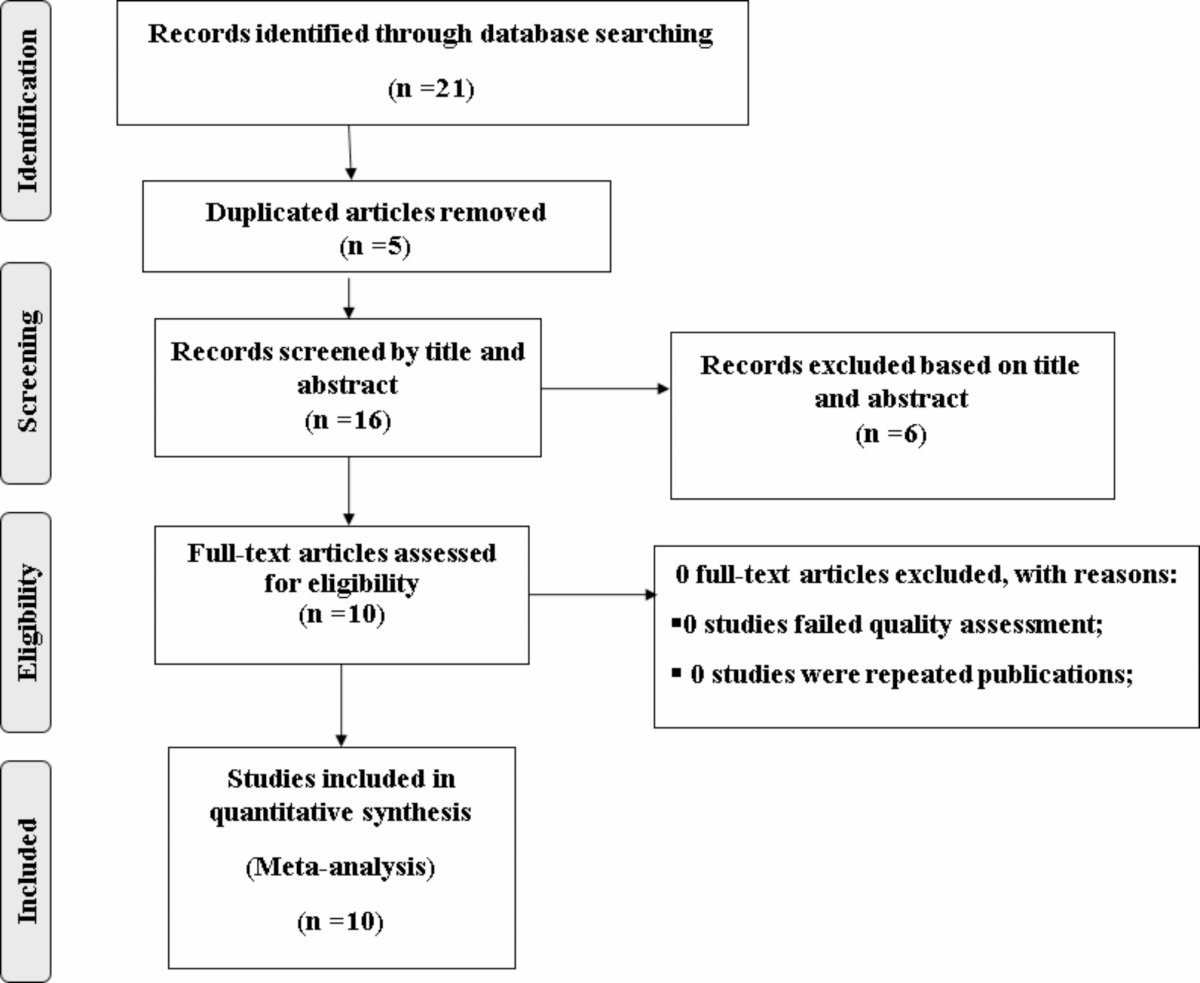
PRISMA flow diagram of the included studies for the systematic review and meta-analysis of Prevalence and associated factors of Early Initiation of Sexual Intercourse among Youth in Ethiopia

This review included published and unpublished studies on the Prevalence and associated factors of Early Initiation of Sexual Intercourse among Youth in Ethiopia. A total of 21 records were identified through electronic database searching. Five duplicated records were removed, and the remaining articles were excluded using their titles and abstracts. Ten full-text articles were evaluated for eligibility (Fig. 1).

## Characteristics of included studies

All studies included in this systematic review and meta-analysis were cross-sectional. The sample size of the studies ranged from 326 to 1294. Overall, this systematic review and meta-analysis included a total of 6355 study participants. The studies were carried out from 2008 to 2022 in different parts of Ethiopia (Table 1).

**Table 1:** Summary characteristics of studies included in the systematic review and meta-analysis

Authors	Study year	Study design	Sample size	Response rate	Prevalence	Quality score
marelign et al.	2013	cross-sectional	405	100	52.5	90.5%
fekadu et al.	2008	cross-sectional	1294	95.5	66	84.5%
Tewodros et al.,	2018	cross-sectional	453	100	17.9	88.5%
Eskezaw et al.	2016	cross-sectional	723	99.3	18.4	74.5%
Biruk et al.	2016	cross-sectional	640	100	38	60.5%
mulu et al.	2016	cross-sectional	613	100	66.6	77.5%
Samuel et al.	2020	cross-sectional	604	99.8	21.1	79%
Tigist et al(unpublished)	2022	cross-sectional	622	96	42	62.6%
Digafe et al(unpublished)	2021	cross-sectional	675	100	60	60.5%
Habtamu et al(unpublished)	2014	cross-sectional	326	98.2	69	72.5%

### Prevalence of early initiation of sexual intercourse

**Fig. 2:**
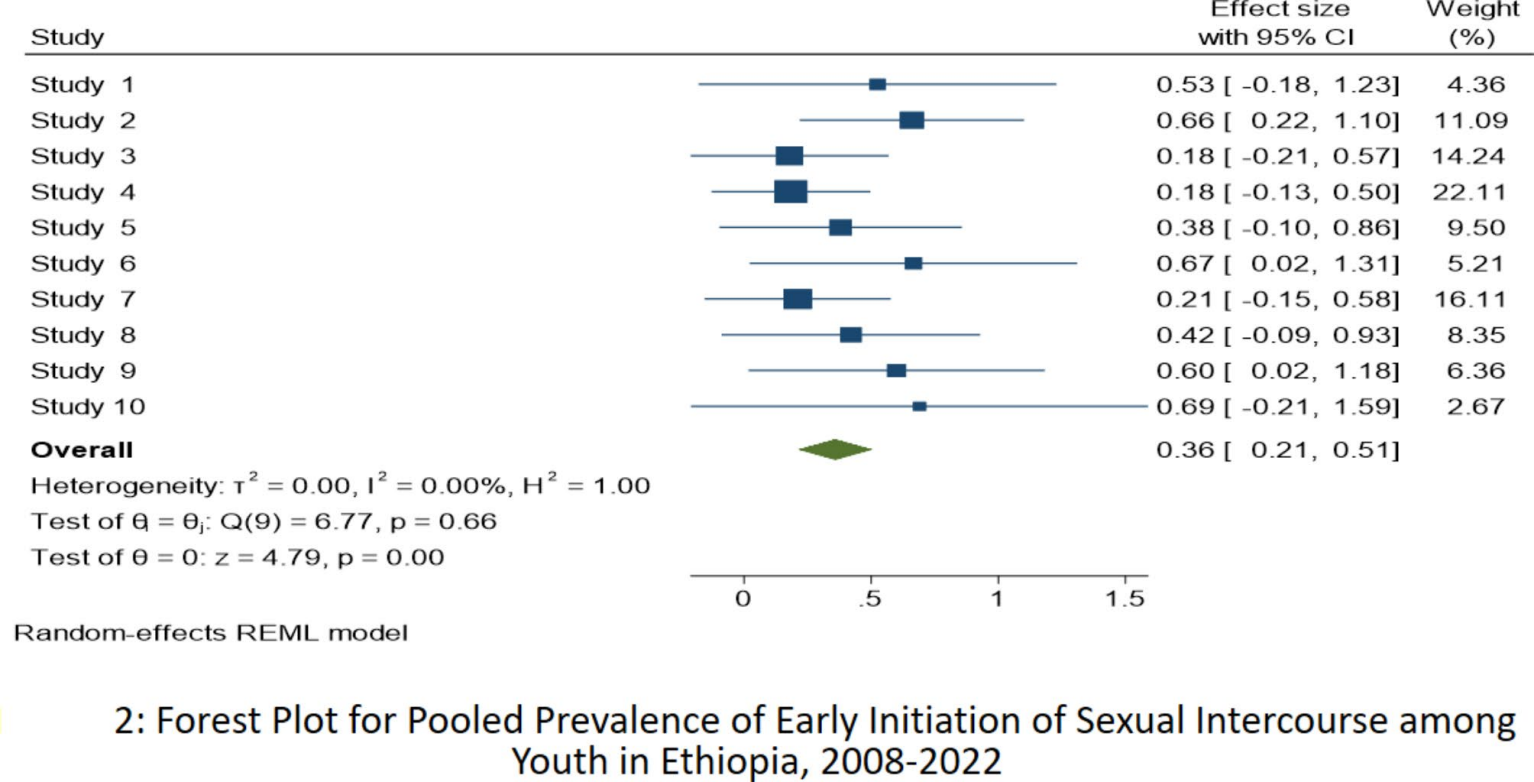
Forest Plot for Pooled Prevalence of early initiation of sexual intercourse among Youth in Ethiopia, 2008–2022

**Fig. 3:**
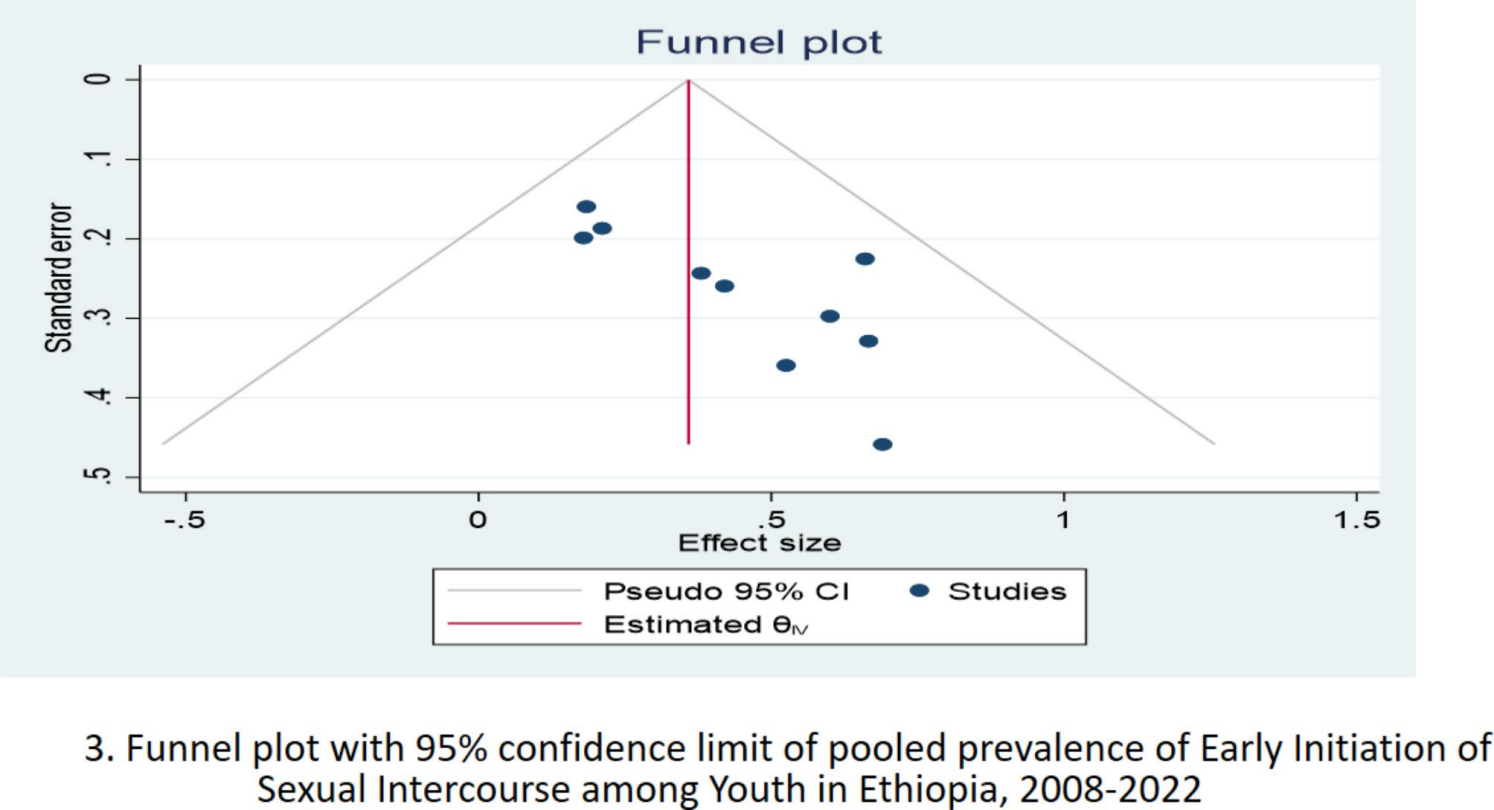
Funnel plot with 95% confidence limit of pooled prevalence of Early Initiation of Sexual Intercourse among Youth in Ethiopia, 2008–2022

Pooled analysis was conducted using the inverse-variance fixed-effects model since there was no evidence of detected heterogeneity, where I^2^ = 0.0%, p < 0.001, Cochran’s *Q* statistic k = 9, p-value = 0.661, and Forest Plot (Fig. 2). The pooled prevalence of early initiation of sexual intercourse among youth in Ethiopia was 36.0% (95%CI: 21.0, 51.0). However, due to the presence of publication bias trim and fill method of analysis was done to correct for funnel plot asymmetry and adjust the final pooled estimate. Therefore, the final adjusted pooled prevalence of early initiation of sexual intercourse was 24.7% (95%CI: 10.4, 38.9). The funnel plot also revealed an asymmetrical appearance (Fig. 3). Furthermore, Egger’s regression asymmetry test indicated significant publication bias, p-value = 0.0447, and Begg rank correlation tests, p-value = 0.0491.

### Pooled associated factors of early initiation of sexual intercourse

This meta-analysis indicated that the sex of the youth, knowledge of HIV/AIDS prevention, Alcohol use, khat chewing, View of pornographic films, cigarette smoking, and poor family controls were the pooled factors of early initiation of sexual intercourse among Youth in Ethiopia. Being female is three times more likely to practice early initiation of sexual intercourse as compared to males (AOR = 3.57; 95% CI: 1.387,5.743), Those who have poor knowledge of HIV/AIDS prevention 35% times more likely to have early initiation of sexual intercourse than good knowledge (AOR = 3.65; 95% CI: 1.981,5.309). Those who drank alcohol were two times more likely to have early initiation of sexual intercourse compared to those who did not use alcohol (AOR = 2.05; 95% CI: 1.415, 2.679). Khat chewer was three times more likely to have early initiation of sexual intercourse as compared to not chewer (AOR = 3.03; 95% CI: 1.800, 4.254). The odds of early initiation of sexual intercourse for youth who were Viewed pornographic films about 4 times higher than those who did not view pornographic films (AOR = 4.21, 95% CI: 2.135, 6.283). Cigarette smokers were two times more likely to have early initiation of sexual intercourse as compared to non-smokers (AOR = 2.74; 95% CI: 2.102, 3.370). Poor family controls were 39% times more likely to have early initiation of sexual intercourse compared to good family controls (AOR = 4.39; 95% CI: 2.572, 6.199)(Table 2).

**Table 2:** Pooled associated factors of Early Initiation of Sexual Intercourse in Ethiopia, 2008–2022

Variables	Categories	Pooled AOR (95% CI)	Heterogeneity		
			I^2^	Q statistic	p-value
Sex	Female	3.57(1.387 ,5.743)	99.8%	1651.67	0.000
	Male	1			
knowledge on HIV	Poor	3.65(1.981,5.309)	99.9%	2300.78	0.000
	Good	1			
Alcohol use	Yes	2.05(1.415, 2.679)	98.8%	427.96	0.000
	N0	1			
khat chewing	Yes	3.03(1.800, 4.254)	99.1	652.56	0.000
	No	1			
View pornographic	Yes	4.21(2.135, 6.283)	99.6%	1373.28	0.000
	No	1			
cigarette smoking	Yes	2.74(2.102, 3.370)	92.0%	49.78	0.000
	No	1			
poor family controls	Yes	4.39(2.572, 6.199)	99.5%	369.62	0.000
	No	1			

## Discussion

Early initiation of sexual intercourse is a risk factor for teenage pregnancy and contracting HIV and other sexually transmitted infections. This study aimed to estimate the pooled prevalence and associated factors of early initiation of sexual intercourse among Youth in Ethiopia. Based on the findings, the pooled prevalence of early initiation of sexual intercourse among Youth in Ethiopia was 24.7% (95%CI: 10.4, 38.9).

This finding is in line with a study done on teenagers in the United States [31], China [32], Busan, Korea [33], sub- Saharan Africa [34]. These findings could suggest that the issue of early sexual initiation among youth is a common public health concern among young people worldwide, regardless of differences in socio-cultural settings. but it is lower than the study done in Brazil [35]. The possible discrepancy might be due to the reason for early sexual initiation in the study area being the sociocultural activities that are highly valued and practiced among youths in that specific area which may not be the case in the former studies with low prevalence.

In this study, female is more likely to practice early initiation of sexual intercourse as compared to male. This study was consistent with studies conducted in different areas [20, 36, 37]. It is because of cultural constraints that females are often given less power to resist the pressure associated with sexual relationships. Although females mature earlier than males physiologically, physical maturation alone may not be accountable for early sexual activities. The sociocultural beliefs and attitudes of society towards early sex, along with the limited decision-making power of young girls, maybe the reasons for early sexual initiation in the study area and beyond. It is against the study conducted in Busan, Korea [33]. This may be due to cultural norms that encourage and approve of the sexual experimentation of males and the value given to virginity for females.

The current finding revealed that those smokers and alcohol drinkers commence sex at an earlier age and the result was consistent with other studies in Busan, Korea [33], and South Africa [38]. This study was also in line with a study done in Ghana [39]. This might be due to the effect of substances which alters the healthy thinking ability of the youth and results in unplanned and unsafe sex.

Youths who chewed khat were also found to be more likely to initiate sexual intercourse earlier than their counterparts. This finding was consistent with previous studies [16, 22]. These circumstances may arise because beer houses generally offer chances to find casual partners. Additionally, youths are more likely to engage in unprotected sex after chewing chat because it diminishes their self-control and sexual negotiation skills [40]. Another possible explanation for this implication could be due to a change of track of mind tempted by chat chewing which stirred them to have early sexual initiation [41].

Youths who have poor knowledge of HIV/AIDS prevention are more likely to practice early initiation of sexual intercourse than good knowledge. This is similar to the study findings from Nigeria [42]. The possible reason for this might be that knowledge about HIV transmission will enable them to avoid early sexual intercourse; because they know the consequences of early initiation of sexual intercourse which includes HIV/AIDS.

In the current study, the odds of early initiation of sexual intercourse for youth who were Viewed pornographic films higher than for those who did not view pornographic films. This finding was in line with other studies conducted [17, 43]. This could be because pornographic materials can stimulate psychological and mental sexual desire and empress to experiment with what has been observed. The impulsive nature of pornographic materials leads to erotic sexual stimulation or early sexual practice [44, 45].

Youth who had poor parental monitoring were more likely to start sexual activity early than those who had good parental monitoring. This is consistent with studies done in Kenya [46]. It is supported by these discoveries that sexual relationships could serve as a substitute measurement for familial proximity in the absence of the latter [47]. A possible contributing element could be the comparatively inadequate monitoring by parents in households with single parents. There exists a connection between parental supervision and the composition of a family [48, 49].

The strengths of this study; Systematic review and meta-analysis methodologies were used, which allowed the researchers to synthesize data from multiple studies to provide a more comprehensive understanding of early initiation of sexual intercourse among Ethiopian youth and the study has a rigorous inclusion and exclusion criteria, which ensures the quality of the studies included in the analysis. This study also has some limitations; The study is limited to studies published in English and could have missed studies conducted in other languages, which may have been relevant to the study, and the studies included in the review often had different definitions of “early initiation” of sexual intercourse, which may have contributed to variations in the prevalence estimates.

## Conclusions

The pooled prevalence of early initiation of sexual intercourse among Youth in Ethiopia was high. Being female, poor knowledge of HIV/AIDS prevention, alcohol use, khat chewing, Viewing pornographic films, Cigarette smoking, and poor family controls were associated factors of early initiation of sexual intercourse. It is recommended that targeted interventions be put in place to address the high prevalence of early initiation of sexual intercourse among youth in Ethiopia. These interventions should focus on addressing the associated factors such as poor knowledge of HIV/AIDS prevention, alcohol use, khat chewing, viewing pornographic films, cigarette smoking, and poor family controls. It is important that these interventions are gender-sensitive and take into consideration the unique challenges faced by females in accessing sexual and reproductive health services.

## Data Availability

The datasets used and/or analyzed during the current study available from the corresponding author on reasonable request.

## Abbreviations

AOR: Adjusted Odds Ratio
CI: Confidence Interval
EDHS: Ethiopian Demographic and Health Survey
HIV: Human Immune virus
JBI: Joanna Briggs Institute
STIs: Sexually transmitted infections
